# HIV testing and prevention among foreign-born Men Who have Sex with Men: an online survey from Sweden

**DOI:** 10.1186/s12889-016-3992-y

**Published:** 2017-01-31

**Authors:** Susanne Strömdahl, Fredrik Liljeros, Anna Ekéus Thorson, Kristina Ingemarsdotter Persson, Birger C. Forsberg

**Affiliations:** 1 Department of Public Health Sciences, Karolinska Institutet, Tomtebodavägen 18a, floor 4, SE-171 77 Stockholm, Sweden; 2 Department of Sociology, Stockholm University, SE-106 91 Stockholm, Sweden

## Abstract

**Background:**

There is an increasing trend toward international migration worldwide. With it comes a challenge for public health and public funded health care systems to meet the migrating population’s health needs. Men who have sex with men are a key population for HIV, contributing an estimated 42% of new HIV cases in Europe in 2013. HIV monitoring data suggest that foreign-born MSM are not only exposed to a high risk of HIV before migration but also while living in Sweden. The aim of this study is to examine HIV testing prevalence and uptake of HIV prevention interventions among foreign-born MSM living in Sweden.

**Methods:**

A web survey available in English and Swedish was conducted from October 1 to October 30, 2013 via a Scandinavian Web community for Lesbian, Gay, Bisexual, Transgender and Intergender people. The web survey included modules on sociodemographics, condom use, sexual risk behaviour and HIV/STI testing experience. 244 eligible MSM participants born abroad and living in Sweden participated in the study. Descriptive and inferential analysis was performed.

**Results:**

Half of the foreign-born MSM participants in this study had been tested for HIV during the last 12 months. Participants who had lived in Sweden less than or equal to 5 years were more likely to have been tested for HIV during the last 12 months. Having talked about HIV/STI with a prevention worker during the past year was associated with having been tested for HIV. Requested services among the majority of participants were HIV rapid test, anonymous HIV testing, HIV/STI testing outside of the health care setting and MSM-friendly clinics.

**Conclusion:**

Efforts are needed to promote HIV testing among foreign-born MSM. Peer outreach, individual and group counselling may be preferred interventions to do so. In addition, it is critically important to increase HIV testing among foreign-born MSM who have lived in Sweden for more than five years. Further research should explore if scale up of implementation of requested services may increase frequency of HIV testing and detection of new cases linked to treatment among foreign-born MSM living in Sweden.

## Background

Human migration has increased globally during the last decade. In 2013, 232 million people were estimated to be migrants, of whom 7% were estimated to be refugees. 72 million migrants resided in Europe 2013, of whom the majority were born in other European countries [1]. The United Nations (UN) defines a migrant as a person who changes his/her country of residence [1]. Sweden’s population of 9.8 million included an estimated 1.6 million international migrants in 2014 [2]. About two-thirds of migrants globally travel from a low-income country to a high-income country [3]. Social and political factors also force people to migrate. One of these factors is that in parts of the world people are persecuted for their sexual behaviour as men who have sex with men (MSM) [4]. A current and future challenge for public health and public funded health care systems in high-income settings is to meet the migrating population’s health needs.

MSM is a key population for HIV worldwide, contributing 42% of estimated new HIV cases in Europe in 2013 [5, 6]. In addition, 35% of newly diagnosed HIV infections in 2013 were reported among migrants in Europe [7]. A recent study reports that the majority of migrants diagnosed with HIV within the last 5 years, living in Spain, the UK, Belgium, Portugal, Greece, Switzerland, the Netherlands, Italy and Germany probably acquired the disease in their current country of residence rather than their birth country [8]. Migrants in Europe also have a higher risk of late diagnosis of HIV than non-migrants [9].

HIV prevalence among MSM in Sweden was estimated at 2–6% in 2012 in comparison to 0.06% in the general population [10]. Between 2010–14, approximately 130 MSM per year were newly reported to be diagnosed with HIV in Sweden. Half of these are foreign-born MSM, which is an increase compared to 40% during the previous 5 year period (2005–2009) [11]. The proportion of newly diagnosed HIV cases among foreign-born MSM resulting from sex between men while in Sweden (as reported by the man himself) was 26% between 2005–2009 and increased to 36% between 2010–2014 [11]. This indicates that foreign-born MSM are not only exposed to a high risk of HIV before migration but also while living in Sweden.

Sexual risk behaviour among MSM in Sweden has been studied in three earlier web-based banner surveys, a form of online convenience sampling, in 2006, 2008 and 2010 [12–14]. The two latter surveys included 15% and 18% foreign-born MSM [12, 14]. The 2008 study compared sexual risk behaviour for HIV/STI between MSM born in Sweden and abroad and found no difference in this particular convenience sample of MSM [14]. Other possible reasons for the elevated risk of acquiring HIV while in Sweden among foreign-born MSM is that they may be sexually active within a network where HIV/STI prevalence is higher [15]. Another reason could be that due to low uptake of regular HIV/STI-testing those persons may be undiagnosed while living with asymptomatic HIV/STI for longer time periods during which they are unaware of the risk of transmission.

Barriers to HIV testing among migrant MSM in the UK are reported to include fear of HIV, fear of legal consequences if living with HIV, stigma and discrimination, lack of culturally sensitive and competent services, language difficulties, lack of knowledge of health services and low priority given to HIV [16]. Foreign-born MSM who have lived for a long time in Sweden may overcome some of these barriers such as language, knowledge of health services and familiarity with rights, while other barriers such as stigma may remain.

The MSM population in Sweden and elsewhere lacks a sampling frame, which makes probability sampling difficult. Sampling strategies to recruit MSM includes respondent driven sampling within the social network of MSM [17]. However, foreign-born MSM are a heterogeneous group and not likely to be interconnected in merely one social network, rather several interlinked social networks. Another strategy is time location sampling from venues where foreign-born MSM meet, which could recruit foreign-born MSM living in metropolitan areas [18]. As foreign-born MSM represents a diverse group it is necessary to complement sampling strategies and studies to capture different parts of the population.

In conclusion, data suggest that the number of foreign-born MSM living in Sweden newly diagnosed with HIV is increasing. Improved knowledge of HIV-testing behaviour in this group is needed to ensure antiretroviral treatment for those living with HIV, which in addition is proven to lower HIV incidence by lowering community viral load [5]. The aim of this study is to examine HIV-testing prevalence and uptake of HIV prevention interventions including different HIV-testing options among foreign-born MSM living in Sweden.

## Methods

### Study population, recruitment and setting

A web survey, MSM 2013, available in English and Swedish was conducted from October 1 to October 30, 2013 via a Scandinavian Web community for Lesbian, Gay, Bisexual, Transgender and Intergender (LGBTI) people [19]. A detailed description of the study recruitment is available elsewhere [20]. Eligibility was defined by being registered as living in Sweden, 15 years or older and not registered as a woman on the Web community. Out of 52,979 eligible Web community members 14,514 were selected by stratified random sampling by age and county of residence to provide a sample that was representative of the Web community population. 2,751 MSM participated, of whom 289 reported being born abroad. Forty-five adopted MSM who were born abroad were excluded because they do not share the experiences of foreign-born migrants, having arrived in Sweden at a later age. This sampling strategy thus provided a sample of 244 MSM participants who are born abroad and live in Sweden which are included in the data analysis.

### Web survey design

The questionnaire was developed by a multidisciplinary team including researchers, representatives of the Swedish Public Health Agency and seven different NGOs representing the MSM community. The web survey included modules on sociodemographics, condom use, sexual risk behaviour and HIV/STI testing experience. The survey was piloted with eleven MSM informants to ensure appropriateness, simple language and user friendliness.

### Statistical analysis

The achieved sample of 244 foreign-born MSM is treated as a convenience sample in the data analysis. Descriptive and inferential data analysis was performed using SPSS Statistics v.22. Chi-square and *t*-test analyses were performed regarding sociodemographics, sexual identity and practice variables.

### Variables definitions

Age was divided between young (<25), middle age (25–40) and older (>40) men as previous findings show that the middle group test more frequently [12]. The majority of participants (57%) had tertiary education and the education variable was dichotomized between tertiary education and lower. The dividing point for the number of years lived in Sweden was 5 years [21]. Participants having lived in Sweden for 5 years or less may be targeted by health interventions for newly arrived immigrants, while participants having lived in Sweden longer are not considered to belong to this group. This time frame is therefore of interest. The reason for moving to Sweden was dichotomized between refugees seeking asylum (21%) and other reasons for migration (work, study or joining partner/family). Asylum-seekers in Sweden are offered a set of health services, including HIV testing, which motivated this division [22]. Those having had unprotected anal intercourse (UAI) with a casual sex partner during the last twelve months (40%) were compared to those who had not [23]. Similarly, having a steady sex partner in the past twelve months was divided between those with (50%) and without. Participants having had unprotected sex with both male and female sex partners during the last twelve months (26%) were compared to those reporting only unprotected sex with men. Knowing where to access testing for HIV on short notice was divided between participants with this knowledge (83%) and those without. Having accessed different prevention services in the last twelve months such as free condoms, online or printed information on HIV/STI and having talked to a prevention worker were deemed to possibly affect the decisions to test for HIV and were therefore included.

### Logistic regression analysis

Variables were analysed by univariate logistic regression and *t*-test to identify factors associated with having been tested for HIV during the last twelve months. Sixteen variables were tested for an association with having been tested for HIV during the last twelve months. Due to collinearity two variables, living with HIV and having tested for other STIs during the last 12 months, were excluded. The variables with a p-value below 0.05 were included in the final model. The Hosmer and Lemeshow test was used to check for goodness of fit. Included variables were checked for interactions, and no significant interaction was found.

### Ethical approval

Ethical approval for this study was obtained from the Regional Ethical Review Board, Stockholm, Sweden (Dnr 2013/248-31/3). Participation was anonymous and no personal identification data were collected. Informed consent was provided by all participants after having read the study information by an active click to confirm participation. Parental consent was not sought for participants as the Swedish regulations regarding ethical trial for research among humans states that the age of consent is 15 years old in Sweden [24]. The consent procedure was approved by the Regional Ethical Review Board, Stockholm, Sweden.

## Results

### Sociodemographic characteristics of the study population

The median age of participants was 36 years, further details are reported in Table 1. Over half (57%) had tertiary education and 67% were employed. Median time spent living in Sweden was 15 years, ranging from less than one year to 58 years. Over half (57%) held Swedish citizenship, 25% had a permanent residency permit and 0.4% was in the process of seeking asylum. Most (58%) were born in Europe, a fifth were born in Asia (20%), about a tenth in South America (11%) and North and Central America (9%) respectively. Lastly, a small numbers of participants were born in Africa (1%).

**Table 1:** Sociodemographic characteristics, sexual orientation and behaviour among participants

Variable		Mean/Median or Percentage	N
Age		38/36 (16–73)	243
Residential area	Stockholm region	26.7%	64/240
	Gothenburg region	12.5%	30/240
	Malmö region	11.3%	27/240
	City with >10 000 inhabitants	37.1%	89/240
	Town or rural region with <10 000 inhabitants	12.5%	30/240
Education	Not completed primary or lower-secondary level education	1.2%	3/244
	Primary or lower-secondary level education	3.7%	9/244
	Upper-secondary education level or vocational training	38.5%	94/244
	University education	56.6%	138/244
Occupation	Employed	68.3%	166/243
	Student	15.6%	38/243
	Unemployed	6.2%	15/243
	Retired	4.9%	12/243
	Long-term sick leave	4.9%	12/243
Years lived in Sweden		18/15 (0–58)	241
Years lived in Sweden by group	0-5	27.8%	67/241
	>5-10	14.1%	34/241
	>10-15	7.9%	19/241
	>15-58	50.2%	121/241
Citizenship status	Swedish citizenship	56.6%	137/242
	Permanent residence permit	25.2%	61/242
	European citizenship	1.2%	3/242
	Nordic citizenship	1.7%	4/242
	Work permit/Student visa	8.7%	21/242
	On-going asylum process	0.4%	1/242
	Other	1.7%	4/242
	Do not want to answer	4.5%	11/242
Reasons for moving to Sweden	To work	22.5%	49/218
	To study	17.4%	38/218
	To be with partner/spouse/cohabitant	15.6%	34/218
	To be with relatives	23.9%	52/218
	To seek asylum	20.6%	45/218
Region of birth	Africa	1.2%	3/244
	Asia	20.1%	49/244
	Europe except Sweden	58.2%	142/244
	North & Central America	9.4%	23/244
	Oceania	0	0/244
	South America	11.1%	27/244
Sexual orientation	Homosexual	63.9%	156/244
	Bisexual	22.5%	55/244
	Heterosexual	2.5%	6/244
	No category/Other	10.2%	25/244
	Don't know	0.8%	2/244
Relationship with	No one	49.6%	120/242
	Man	34.4%	84/242
	Woman	9.4%	23/242
	Several persons of which at least one is a man	6.2%	15/242
No. of sexual partners last 12 months		8.8/3 (0–100)	200
Having had unprotected sex with men and women last 12 months		9.2%	22/240
No. of casual male sex partners for UAI last 12 months		2,5/0 (0–100)	193
Member of LGBTI organisation		36.5%	80/219

### Sexual identity and behaviour

The majority (64%) identified as homosexual as reported in Table 2. Half (50%) reported being single, 35% were in a relationship with a man and 9% with a woman. The median number of sexual partners during the last twelve months was three. About 10% reported having had sex with both men and women during the last twelve months. Frequency of male partners for UAI was on average 2.5, and the median was 0.

**Table 2:** HIV/STI testing experience among participants

Variable		Percentage	N
Most recent HIV test	Last 12 months	45.4%	104/229
	>12 months	32.8%	75/229
	Never	19.7%	45/229
	Don’t remember	2.2%	5/229
Self-reported HIV status	Living with HIV	3.9%	9/228
	Negative	84.2%	192/228
	Uncertain	10.5%	24/228
	Don’t want to answer	1.0%	3/228
Don't know where to get tested for HIV on short notice		17.1%	37/217
Reasons for not testing among those never tested for HIV	I believe that I have not taken any risks	51.1%	23/45
	I don’t know where to get tested	22.2%	10/45
	I’m living in a monogamous relationship	20.0%	9/45
	I’m afraid I will feel like a failure if I have HIV	20.0%	9/45
	I’m afraid that staff or other visitors to the clinic will recognize me	15.6%	7/45
	I’m afraid I will become ill	15.6%	7/45
	Due to the rules in the Swedish Communicable Disease Act	15.6%	7/45
	I’m afraid I will lose family and friends	13.3%	6/45
	I don’t trust confidentiality in the healthcare system	11.1%	5/45
	I don’t want to know my HIV status	11.1%	5/45
	I’m afraid the test results would have a negative influence on my sex life	11.1%	5/45
	Due to how the Swedish penal code is applied to HIV	8.9%	4/45
	I’m afraid I will lose my partner	8.9%	4/45
	It’s difficult for me to get to a clinic	6.7%	3/45
	I’m afraid the test results would affect my chances of staying in Sweden	4.4%	2/45
	I’m afraid I will lose my job	2.2%	1/45
	There is no cure for HIV, so I see no point in getting tested	2.2%	1/45
	I have been denied an HIV test by health personnel, even though I wanted to get tested	2.2%	1/45
Most recent STI test	Last 12 months	37.0%	84/227
	>12 months	26.9%	61/227
	Never	33.0%	75/227
	Don’t remember	3.1%	7/227
Self-reported STI diagnoses last 12 months	Chlamydia	6.3%	9/144
	Gonorrhoea	4.2%	6/144
	Syphilis	0.7%	1/144
	Condyloma/HPV	0.7%	1/144
	Hep A	0.7%	1/144
	Hep B	0.7%	1/144
	Mycoplasma	0.7%	1/144
	Hepatitis C	0	0/144
	Genital herpes	0	0/144
	Lymphogranuloma venereum	0	0/144
	No STI	88.9%	128/144
	Don't know	1.4%	2/144

### HIV/STI testing experience

**Fig. 1:**
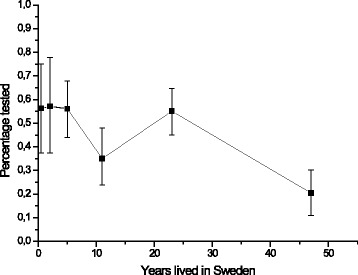
Participants’ experience of having been tested for HIV during the last 12 months in relation to how long they have lived in Sweden

**Fig. 2:**
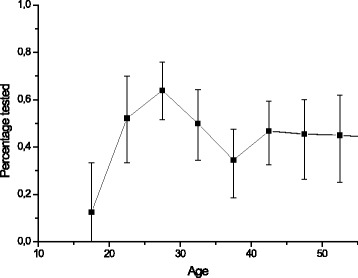
Participants’ experience of having been tested for HIV during the past 12 months in relation to their age

Close to half (45%) had been tested for HIV during the last twelve months, an additional third had been tested at some time and a fifth reported never having been tested for HIV (See Table 2). Participant experience of having tested for HIV during the last 12 months showed a declining trend with increased years of stay in Sweden, as shown in Fig. 1. In addition, participant experience of having tested for HIV during the last 12 months varied with age peaking at 28 year old, as shown in Fig. 2.

Self-reported HIV prevalence among participants was 4%. The majority (88%) felt certain regarding their HIV serostatus, while 11% reported being uncertain. Almost a fifth (17%) reported not knowing where to be tested for HIV. Among those who had never been tested for HIV, the most common reason given was that’I’ve never thought of it’ or the belief that’I don’t take risks’. While a fifth of those who had never been tested reported that they didn’t know where to access HIV-testing, 2% reported having been denied a HIV test by health personnel.

**Fig. 3:**
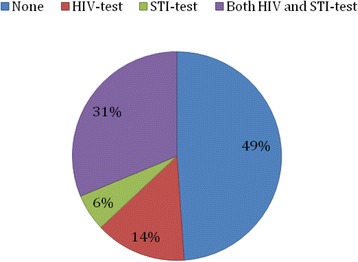
Experience of HIV and STI testing during the past 12 months among participants

37% reported having been tested for STIs during the last twelve months. A third reported never having been tested for STIs. About a third had been tested for both HIV and STIs during the last 12 months as shown in Fig. 3.

### HIV/STI prevention services used and requested by participants

Most participants reported having read online (66%) and printed (54%) information on HIV/STI during the past 12 months. Half (51%) had received free condoms and about a third (37%) had talked about HIV/STI with a prevention worker during the same time period.

The majority (76%) requested a rapid test for HIV and easily accessible condoms and lubricants in places where MSM meet. In addition, there were requests for anonymous HIV testing (69%), MSM-friendly clinics (68%), HIV/STI testing outside of the healthcare setting (63%), vaccination for hepatitis A, B (66%) and HPV (63%) and the availability of condoms and lubricants by mail (63%). Over half (58%) requested web-based HIV/STI information.

About half (48%) requested individual counselling regarding HIV/STI and safe sex, 39% requested online counselling on these issues and 34% group counselling. 38% requested SMS reminder for HIV/STI-testing, 37% requested pamphlets on HIV/STI and safe sex and 31% requested support groups to deal with sexuality and health.

### Associations with having been tested for HIV during the past twelve months

The univariate analysis results are presented in Table 3. The following variables were found to be significantly associated with having been tested for HIV during the last 12 months: lived in Sweden for less than or equal to 5 years (OR 1.87, 95%CI 1.04–3.36), knowledge of where to get tested for HIV (OR 2.21, 95%CI 1.03–4.74), talked about HIV/STI with a prevention worker during the last 12 months (OR 5.33 95%CI 2.94–9.65), received free condoms during the last 12 months (OR 2.23, 95%CI 1.20–3.82), read online about STI/HIV during the last 12 months (OR 2–56, 95%CI 1.42–4.60), and read printed information about HIV/STI during the last 12 months (1.86, 95%CI 1.09–3.19).

After multivariate adjustment, lived in Sweden for less than or equal to 5 years (aOR 2.12 95%CI 1.07–4.18) and talked to a HIV/STI prevention worker during the last 12 months (aOR 4.6 95%CI 1.28–9.29) remained significantly associated with having tested for HIV in the past 12 months.

**Table 3:** Associations with HIV testing during last 12 months among participants

Variable		HIV test during last 12 months	
		Univariate OR (95%CI)	Multivariate OR (95%CI)
Age	<25	0.85 (0.40–1.83)	
	25-40	1.53 (0.91–2.59)	
	<40	0.69 (0.40–1.18)	
Tertiary education vs. lower		1.59 (0.94–2.70)	
Occupation	Employed	0.95 (0.70–1.30)	
	Unemployed	0.70 (0.24–2.06)	
	Long-term sick-leave	0.45 (0.11–1.81)	
	Student	0.74 (0.35–1.56)	
	Retired	0.53 (0.15–1.82)	
Years lived in Sweden < =5 years vs. >5 years		1.87 (1.04–3.36)*	2.12 (1.07–.4.18)*
Seeking asylum as reason for coming to Sweden vs. other reasons for migration		1.18 (0.59–2.38)	
Having had UAI with casual partner in the past 12 months vs. not		0.75 (0.41–1.38)	
Single vs. being in a relationship		0.85 (0.50–1.43)	
Having had unprotected sex with men and women during last 12 months vs. only men		0.88 (0.34–2.28)	
Knowledge of where to get tested for HIV on short notice vs. no such knowledge		2.21 (1.03–4.74)*	2.01 (0.88–4.80)
LGBTI organization member vs. not		0.76 (0.36–1.59)	
Talked about HIV/STI with a prevention worker during last 12 months vs. not		5.33 (2.94–9.65)*	4.6 (2.28– .9.29)*
Received free condoms during last 12 months vs. not		2.23 (1.30-3.82)*	1.46 (0.77-2.76)
Read online about STI/HIV during last 12 months vs. not		2.56 (1.42-4.60)*	1.73 (0.81-3.70)
Read printed information about HIV/STI during last 12 months vs. not		1.86 (1.09-3.19)*	0.71 (0.33-1.51)

* *p*-value <0.05

## Discussion

About half of the foreign-born MSM participants in this study had been tested for HIV during the last twelve months. Participants who had lived in Sweden less than or equal to 5 years were more likely to have been tested for HIV during the last 12 months. Having talked about HIV/STI with a prevention worker during the past year was associated with having been tested for HIV.

Initially, after moving to Sweden, access to HIV testing in an MSM-competent, non-stigmatizing environment may encourage frequent testing. Protected gay rights and experience of the Swedish gay community might enable disclosure as an MSM to health care staff. This may increase the likelihood of being offered HIV testing regularly. In addition, as a person migrates to a new setting, new sexual relationships may take place that motivate the individual to get tested. Easy access to HIV/STI testing is common in urban areas in Sweden, which may increase the frequency of testing [25, 26].

The European MSM Internet Survey (EMIS) was conducted in 2010 as an online banner survey among 3,089 MSM living in Sweden and reported that 30% had been tested for HIV during the last 12 months, in comparison to 45% in this study [12]. As both studies report on convenience samples of MSM, they cannot be compared and conclusions to the wider population of MSM living in Sweden cannot be made. However it is interesting to note that HIV-testing rates during the last 12 months among the foreign-born MSM participants in this study is still slightly higher than the most recent estimates reported among MSM in Sweden mentioned above.

Twenty percent of participants reported never having been tested for HIV. The EMIS 2010 study found that 25% of MSM living in Sweden had never been tested for HIV [12]. Further, a study from Britain report that migrant MSM are more likely to have been tested for HIV than MSM born in Britain [27]. One of the most common reasons given for never having been tested for HIV was low perceived risk. Similar findings have been reported among MSM that had never been tested for HIV in Spain [28]. The perceived low risk may be correct, however, it could also be based on inadequate knowledge of HIV transmission among MSM. As participants reported being men who have sex with men they do belong to the group with the highest number of newly diagnosed cases of HIV per year in Sweden. Further research is needed to understand how risk perception and HIV knowledge affects HIV-testing behaviour within this group.

Participants who had talked about HIV/STI with a prevention worker were more likely to have been tested for HIV, indicating that individual counselling and outreach work have an important role in prevention work for foreign-born MSM. A number of HIV testing services were requested by study participants, increasing access to these services might increase HIV testing rates. HIV rapid test, anonymous HIV testing, HIV/STI testing outside of the health care setting and MSM-friendly clinics were requested by the majority of participants and should be a priority in HIV prevention. Making condoms and lubricants easily available in places where MSM meet may increase condom use. Internet-based HIV/STI information interventions were favoured by most, and this finding is consistent with a previous study from 2012 [29].

Increasing HIV testing is crucial for HIV prevention, decreasing the number of undiagnosed people living with HIV is a priority globally and most important to ensure access to treatment for those living with HIV [5]. Biomedical HIV prevention interventions are serostatus-dependent such as treatment as prevention (TasP), post-exposure prophylaxis (PEP), and pre-exposure prophylaxis (PrEP) [5, 30]. In addition, knowledge of serostatus facilitates tailored behavioural and educational interventions. Delayed diagnosis of HIV is associated with poorer response to antiretroviral therapy and a higher risk for morbidity and mortality [5, 31]. A 45% annual HIV-testing rate among foreign-born MSM may be too low to detect those in need of antiretroviral treatment. Being undiagnosed with HIV for long time periods and unaware of the risk of transmission, may increase the risk of sexual transmission due to not receiving treatment and thereby not achieving viral suppression.

The data reported here have limitations, such as being self-reported, which may introduce reporting bias [32, 33]. Efforts to decrease reporting bias included an easy to answer online questionnaire, which was answered anonymously. The survey was available in Swedish and English thereby excluding foreign-born MSM who were not conversant in those languages. As the survey was conducted online on a Web community, MSM who were not members were not able to participate. Since the participants were not randomly selected, it is not possible to generalize results to wider groups of foreign-born MSM living in Sweden. The survey is of cross-sectional design and causality can therefore not be detected. Nonetheless, the data provide important insights into this heterogeneous, at risk of HIV and hard to reach group. Furthermore, half of the sample had lived in Sweden for fifteen years or longer, and less than one per cent were in the asylum-seeking process. There is a need for further studies to explore health needs among foreign-born MSM who are seeking asylum.

## Conclusions

Efforts are needed to promote HIV testing among foreign-born MSM. Peer outreach, individual and group counselling may be preferred interventions to do so. In addition, it is critically important to increase HIV testing among foreign-born MSM who have lived in Sweden for more than 5 years. Further research should explore if scale up of implementation of the requested services; HIV rapid tests, HIV testing outside of the health care setting, anonymous HIV testing and MSM-friendly clinics, may increase frequency of HIV-testing and detection of new cases linked to treatment among foreign-born MSM living in Sweden.
